# Identification of resistance to *Sugarcane mosaic virus, Sugarcane streak mosaic virus*, and *Sugarcane bacilliform virus* in new elite sugarcane accessions in India

**DOI:** 10.3389/fmicb.2023.1276932

**Published:** 2023-10-20

**Authors:** G. Vamsi Krishna, V. Manoj Kumar, P. Kishore Varma, B. Bhavani, G. Vijaya Kumar

**Affiliations:** ^1^Department of Plant Pathology, Agricultural College, Bapatla, Acharya N. G. Ranga Agricultural University, Guntur, Andhra Pradesh, India; ^2^Department of Plant Pathology, Regional Agricultural Research Station (RARS), Lam, Acharya N. G. Ranga Agricultural University, Guntur, Andhra Pradesh, India; ^3^Department of Entomology, District Agricultural Advisory and Transfer of Technology Centre (DAATTC), Amalapuram, Acharya N. G. Ranga Agricultural University, Guntur, Andhra Pradesh, India; ^4^Department of Crop Physiology, Agricultural College, Bapatla, Acharya N. G. Ranga Agricultural University, Guntur, Andhra Pradesh, India

**Keywords:** mosaic, leaf fleck, resistant sources, SCMV, SCSMV, SCBV, RT-PCR/PCR

## Abstract

Sugarcane mosaic and leaf fleck diseases are significant viral diseases affecting sugarcane crops in India. The use of resistant sugarcane varieties is considered the most economical and effective approach to manage viral diseases, especially in vegetatively propagated crops such as sugarcane. *Sugarcane mosaic virus* (SCMV) and *Sugarcane streak mosaic virus* (SCSMV) are the primary pathogens responsible for mosaic disease in sugarcane-growing regions of India. *Sugarcane bacilliform virus* (SCBV), causing leaf fleck disease, is also often found in mixed infections with mosaic symptoms. The study aimed to identify new sources of resistance by screening sugarcane germplasm for resistance to SCMV, SCSMV, and SCBV. The screening was carried out under high inoculum using the infector row method in both plant and ratoon crops. Out of 129 genotypes tested, only 8 were found to be free of mosaic viruses, indicating a rare occurrence of resistant sources. The study revealed that mosaic disease is widespread, with nearly 95% of tested varieties/genotypes being infected with mosaic viruses. SCMV, SCSMV, and SCBV were detected in 121 out of 129 genotypes using the RT-PCR and PCR assays. Based on their response to the viruses, the tested genotypes were categorized into different resistance grades: highly resistant (grade 1), resistant (grade 2), moderately resistant (grade 3), susceptible (grade 4), and highly susceptible (grade 5). The results of the study provide valuable information about elite resistance resources that can be used for the prevention and control of mosaic disease. These resistant genotypes could also serve as potential donors for mosaic and leaf fleck disease resistance in breeding programs.

## Introduction

Sugarcane is a perennial grass of the genus *Saccharum* and family Poaceae. It is cultivated as a vital field crop for sugar and bioenergy production and is grown in more than 110 countries worldwide, covering about 26.34 million hectares and yielding approximately 1,859.39 million tons. Approximately 80% of the world’s sugar demand is met by this crop. Sugarcane has had a historical presence in Asian farmlands since ancient times and continues to occupy a significant place in agriculture and the economy. Major sugarcane-producing countries include India, Brazil, China, Thailand, Pakistan, Mexico, Colombia, Indonesia, the Philippines, and the United States. India stands out as the second-largest producer, accounting for approximately 20% of the world’s cane production, covering 5.15 M ha^–1^ with a production of 405.39 Mt (Food and Agriculture Organization of the United Nations, 2021). The Asian continent, particularly Southeast Asia, being the origin of the crop, is known for its rich genetic diversity. The economic significance of the crop extends beyond sugar to include products like bagasse, crystal white sugar, power, ethanol, and press mud. It is also considered as a potential energy cane/biofuel crop (Matsuoka et al., 2014). Such a potential crop is threatened by viral, fungal, bacterial, and phytoplasmal diseases. Among these, viral diseases are becoming a major concern, leading to varietal degeneration (Viswanathan, 2016). Viral diseases such as mosaic, yellow leaf, and leaf fleck have been reported in sugarcane-growing regions, impacting cane yield and juice quality (Viswanathan et al., 2018).

Sugarcane mosaic disease is primarily caused by the infection of *Sugarcane mosaic virus* (SCMV), *Sugarcane streak mosaic virus* (SCSMV), and *Sorghum mosaic virus* (SrMV). These viruses infect sugarcane plants and lead to the manifestation of mosaic-like symptoms, which involve the development of irregular patterns of light and dark green on the leaves (Lu et al., 2021). Studies have demonstrated that sugarcane mosaic disease in India is attributed to SCMV and SCSMV infections, either alone or in conjunction (Hall et al., 1998; Hema et al., 1999; Viswanathan et al., 2007). Sugarcane mosaic disease, exhibiting typical “mosaic” symptoms (Krishna et al., 2023b), can significantly reduce the sett germination, photosynthetic efficiency, yield, and quality of sugarcane (Costa and Muller, 1982; Smith et al., 1992; Bagyalakshmi et al., 2019). Infected plants are likely to produce fewer stalks and impact the economic value of the harvest. Throughout history, pandemic outbreaks of sugarcane mosaic disease have led to substantial economic losses in the sugarcane industry. These losses have been severe enough to cause financial difficulties and even bankruptcies for sugar companies around the world (Koike and Gillaspie, 1989; Grisham, 2011). Higher yield losses arising due to the severe incidence of this disease led to the discontinuation of several sugarcane cultivars (Singh et al., 1997). Developing sugarcane varieties that are resistant to viruses and rational planting is a key strategy to prevent and control the disease.

*Sugarcane streak mosaic virus* and SCMV have a broad host range that includes not only sugarcane but also other grass hosts like maize and sorghum, making viruses more adaptable and capable of infecting different plant species (Viswanathan et al., 2018). In India, the incidence of mosaic disease is approximately 100%. This widespread prevalence contributes to a significant yield reduction in elite sugarcane cultivars ranging from 30 to 80% (Singh et al., 2003; Viswanathan and Balamuralikrishnan, 2005; Rao et al., 2006). Mixed infections involving SCMV, SCSMV, and *Sugarcane bacilliform virus* (SCBV) have been reported in India. These mixed infections can lead to more severe symptoms, such as dwarfing and necrosis, resulting in substantial production losses for susceptible sugarcane cultivars (Hema et al., 2003; Singh et al., 2009). Severe infections of SCBV in susceptible sugarcane cultivars have been associated with biomass production losses of 25–35% (Comstock and Lockhart, 1996; Lockhart and Autrey, 2000). SCMV is transmitted by various aphid species in a non-persistent manner, making it challenging to control the spread of the virus in the field (Hassan et al., 2003). Developing resistant sugarcane varieties can provide a significant resource for minimizing the damage caused by SCMV and related viruses.

Identification and cultivating resistant varieties are the only available options with respect to the management of viral diseases in sugarcane. Although using virus-free planting material lessens the damage that viruses do to plant crops, ratoon crops experience more severe damage as a result of viral transmission through vectors under field conditions. Therefore, in order to control the disease through host resistance, it is crucial to identify resistant sources from the parents and wild germplasm of sugarcane. Host plant resistance is the ultimate tool to manage viral diseases in vegetatively propagated crops like sugarcane. Few researchers have been working on identifying the resistant sources for sugarcane mosaic disease. Recently, Bagyalakshmi and Viswanathan (2021) screened 210 genotypes, with an emphasis on developing a mosaic scoring system. Furthermore, researchers have primarily confined to a few available cultivars, aiming to identify a resistant source for the sugarcane mosaic and leaf fleck diseases. This study examined the germplasm of sugarcane, including commercial hybrid varieties, genotypes, and recently released varieties, for their symptom manifestation under high inoculum pressure for two consecutive years.

## Materials and methods

### Planting material

A total of 129 sugarcane genotypes were planted in the experimental field of the Division of Plant Pathology at Regional Agricultural Research Station (RARS), Anakapalli, for the evaluation of mosaic and leaf fleck resistance during the period 2021–2022 (plant crop) and 2022–2023 (ratoon crop) crop growing seasons by the infector row method. Entries were planted in two rows of 5 m in length, and 25 three-budded setts were planted in each row. The experiment was laid out in an augmented block design with a susceptible check, 2009A 107, repeated after three entries. Resistance in all the entries was confirmed through RT-PCR and PCR at the formative and maturity stages. Entries that were found to be highly resistant under field conditions in plant crops were planted in a glass house in the next season along with the susceptible check and were inoculated with sugarcane mosaic-infected sap 45 days after planting. Furthermore, resistance was confirmed with RT-PCR and PCR.

### Symptomatology and disease index

The list of 129 sugarcane genotypes evaluated for mosaic and leaf fleck resistance studied in the present study is given in Table 1. The test genotypes were artificially inoculated with the sap of mosaic-infected sugarcane leaves, tested with RT-PCR and PCR assays for the presence of viruses causing mosaic and leaf flecks, and monitored periodically for the manifestation of symptoms typical of the diseases. The types of visual symptoms expressed by the genotypes were recorded from April to November during both crop seasons, namely 2021–2022 and 2022–2023 plant crop and ratoon, respectively. The disease index was recorded in 129 sugarcane genotypes. Throughout the study period, the sugarcane genotypes were consistently monitored for the manifestation of mosaic disease symptoms. The types of visual symptoms displayed by each genotype were carefully recorded and documented. Observations on mosaic incidence were recorded, and percentage incidence was calculated using the below-mentioned formula:


$$\mathrm{Mosaic}\mathrm{incidence}\left(\%\right)=\frac{\mathrm{Number}\,\mathrm{of}\,\mathrm{infected}\,\mathrm{plants}}{\mathrm{The}\,\mathrm{total}\,\mathrm{number}\,\mathrm{of}\,\mathrm{plants}\,\mathrm{observed}}\times 100$$

**TABLE 1:** List of 129 sugarcane genotypes used for the field evaluation for mosaic and leaf fleck disease resistance during 2021–2022 and 2022–2023 crop growing seasons.

S. no.	Accession no.	S. no.	Accession no.	S. no.	Accession no.	S. no.	Accession no.	S. no.	Accession no.
1	2017A 553	27	2018A 8	53	2016A 379	79	2010A 229	105	BO 91
2	2017A 36	28	2018A 13	54	2016A 381	80	CoA 7602	106	Co J 64
3	2017A 65	29	2018A 30	55	2016A 385	81	Co 86032	107	Co7717
4	2017A 103	30	2018A 31	56	2016A 395	82	98A 163	108	Co S 767
5	2017A 191	31	2018A 33	57	2016A 580	83	2000A 225	109	Baragua
6	2017A 196	32	2018A 55	58	2016A 592	84	Co 997	110	Co 62399
7	2017A 205	33	2018A 212	59	2016A 600	85	97A 85	111	Co 86002
8	2017A 253	34	2018A 107	60	2016A 664	86	2000A 56	112	CoS 8436
9	2017A 268	35	2018A 122	61	2016A 672	87	2001A 63	113	Co Se 95422
10	2017A 340	36	2018A 130	62	2015A 301	88	2003V 46	114	Co 09022
11	2017A 405	37	2018A 141	63	2015A 308	89	2003A 255	115	83V 15
12	2017A 408	38	2018A 152	64	2015A 309	90	2004A 55	116	Co 15024
13	2017A 416	39	2018A 144	65	2015A 311	91	Co 7219	117	Co 15026
14	2017A 457	40	2018A 157	66	2015A 333	92	2006A 64	118	Co 15027
15	2017A 497	41	2018A 161	67	81A 99	93	2006A 102	119	Co 12029
16	2017A 68	42	2018A 166	68	93A 145	94	2006A 223	120	Co 13034
17	2017A 73	43	2018A 171	69	97A 85	95	2010A 229	121	Co 14034
18	2017A 94	44	2018A 174	70	2000A 56	96	CoA 7602	122	CoS 08279
19	2017A 187	45	2018A 175	71	2001A 63	97	Co 86032	123	87A 298
20	2017A 236	46	2018A 185	72	2003V 46	98	98A 163	124	Co Lk 14203
21	2017A 269	47	2018A 188	73	2003A 255	99	2000A 225	125	Co 86002
22	2017A 313	48	2018A 190	74	2004A 55	100	Co 997	126	Co Lk 15206
23	2017A 351	49	2018A 195	75	Co 7219	101	CoC 671	127	Khakai
24	2017A 429	50	2018A 196	76	2006A 64	102	Co 419	128	CoC 19337
25	2017A 517	51	2018A 202	77	2006A 102	103	Co 975	129	2009 A 107*
26	2018A 6	52	2018A 203	78	2006A 223	104	Co 1148		

*Susceptible check.

Based on the disease incidence, tested genotypes were categorized according to Li et al. (2018) and mentioned below.

**Disease index scale for mosaic disease of sugarcane**

**Table:** 

Grade	Disease index (%)	Reaction
1	0.00	Highly resistant (HR)
2	1–10.0	Resistant (R)
3	10.1–33	Moderately resistant (MR)
4	33.1–66	Susceptible (S)
5	66.1–100	Highly susceptible (HS)

### Artificial inoculation

The genotypes that were found to be highly resistant in plant crops were planted in a glass house and mechanically inoculated with crude sap from RT-PCR-determined SCMV and SCSMV and PCR-determined SCBV-infected sugarcane leaves extracted in 0.1 M phosphate buffer, pH 7.0 (Użarowska et al., 2009).

### Detection of *Sugarcane bacilliform virus*

#### Total DNA extraction

Total DNA was extracted from the samples collected by following the CTAB technique (Doyle and Doyle, 1987). Leaf samples (each 100 mg), kept at −86°C, were ground to a fine powder using liquid nitrogen in a mortar and pestle. CTAB buffer of about 1 ml was added to the finely powdered samples in a 1.5 ml sterile microcentrifuge tube. The samples were then incubated in a water bath at 65°C for 45 min and allowed to cool to room temperature, followed by centrifugation at 4°C for 10 min at 12,000 rpm. The supernatant was collected in a fresh microcentrifuge tube, followed by the addition of chloroform and isoamyl alcohol (24:1). Following a thorough mixing process, tubes were centrifuged at 4°C for 10 min at 12,000 rpm. Three layers were formed, i.e., upper aqueous phase, middle protein phase, and lower organic phase, among the three upper aqueous phases, which included DNA, and were moved into a fresh microcentrifuge tube, followed by the addition of 0.6 volume of isopropanol, and mixed by gentle inversion for 4–5 times. Later, DNA was allowed to precipitate at 4°C for 30 min. After that, tubes were centrifuged for 10 min at 12,000 rpm to form pellets. The pellet was then centrifuged at 4°C for 10 min at 12,000 rpm with 75% ethanol. The pellet was retained, and the supernatant was discarded. The pellet was then allowed to dissolve in 40 μl of sterile distilled water after being air-dried.

**Primers used for PCR amplification**

**Table:** 

Virus	Region	Synthetic oligonucleotide primers (5′–3′)	References
SCMV	Coat protein	CCCGAAGCTTGCTGG AACAGTCGATGCAGG	Viswanathan et al., 2010
		ATCGCGGCCGCTTA GCCAGCTGTGTGTCTCT	
SCSMV	Coat protein	GGATCCGGACAAGGAA CGCAGCCAC	Viswanathan et al., 2010
		AGATCTCGCACGTC GATTTCTGCTGGTG	
SCBV	RT/RNase H	GCRCCWGCAGTVTT YCARAGGAAGATG	Krishna et al., 2023a
		CCAYCTGATCTCH GAAGGYTTRTG	

#### PCR amplification

The most conserved RT/RNase H domains and sequences have been widely employed in Badnavirus taxonomy and viral detection. Primers specific to SCBV were used in the PCR reaction to target the RT/RNase H coding region of the genome, which is a region of 794 bp in size. A PCR reaction of 25 μl includes 2 μl of DNA, 2 μl Taq buffer, 2 μl Mgcl_2_, 2 μl dNTPs, 10 pmol forward and reverse primer, 1.25 units Taq DNA polymerase (Genei, Bangalore), and sterile Milli-Q water. A thermocycler (Mastercycler, Eppendorf) was used to carry out the PCR reaction at an annealing temperature of 59°C for 45 s. The PCR products were separated by electrophoresis and visualized under a gel documentation system (Vilber E: Box, UK).

### Detection of *Sugarcane mosaic virus* and *Sugarcane streak mosaic virus*

#### Total RNA extraction

All the samples collected from 129 sugarcane genotypes at vegetative and grand growth stages were subjected to RNA extraction. In total, 100 mg of fresh sample was powdered using liquid nitrogen to ensure rapid and thorough disruption of cellular structures. The powdered sample is then transferred to a microcentrifuge tube treated with DEPC and mixed with 1 ml of TRI reagent. The sample and TRI reagent mixture are vigorously shaken to ensure proper mixing and then incubated at 4°C. This allows time for the nucleoprotein complexes to dissociate and for RNA to be released from the cells. After incubation, the mixture is centrifuged at a high speed of 12,000 rpm and kept at a low temperature (4°C) for 10 min. The supernatant (liquid phase) containing the RNA was transferred to a new tube, and 200 μl of chloroform was added. The mixture was shaken to create an emulsion and then incubated at room temperature. This step helps to separate different phases within the solution. The mixture was centrifuged again at 12,000 rpm and 4°C for 15 min. This step leads to the separation of the solution into three distinct phases: a lower organic phenolic phase containing proteins, an inter-phase containing DNA, and an upper colorless aqueous phase containing RNA. The upper aqueous phase, which contains the RNA, is carefully transferred to a new tube. Then, 500 μl of isopropanol is added to the RNA-containing solution, causing the RNA to precipitate out of the solution. The mixture is centrifuged again to form a pellet and washed thrice with ethanol. The pellet is air-dried for 10 min to remove any residual ethanol. Then, the RNA pellet is dissolved in 40 μl of molecular-grade water (Chomczynski and Sacchi, 1987; Kumar et al., 2023). The resulting RNA solution was stored at −86°C for further analysis. The quality of the extracted RNA was assessed by running it on a 1.5% agarose gel, which can provide information about the integrity and concentration of the RNA sample.

#### cDNA synthesis and PCR amplification

The first-strand cDNA synthesis kit (Thermo Scientific) was used for the synthesis of cDNA. The reverse transcription was performed in a 10 μl reaction mixture that contained 2 μl 10 mM dNTPs, 4.375 μl ddH_2_O, 2 μl RT buffer, 0.25 μl Oligod (T), 0.125 μl RT enzyme, 0.25 μl RNase inhibitor, and 2 μl RNA template. The reaction mixture was incubated at 70°C for 5 min, followed by inactivation of the enzyme at 37°C for 90 min, and termination of the reaction at 72°C for 10 min. A PCR reaction volume of 25 μl consisting of 2 μl of cDNA, 2 μl of Taq buffer, 2 μl of Mgcl_2_, 2 μl of dNTPs, 10 pmol of both forward and reverse primers, 1.25 units of Taq DNA polymerase (Genei, Bangalore), and sterile Milli-Q water makeup to the final volume. Thermocycler (Mastercycler, Eppendorf) was used to carry out the PCR reaction with an annealing temperature of 65°C for 45 s. The PCR products were separated by electrophoresis and visualized under a gel documentation system (Vilber E: Box, UK).

### Phylogenetic tree analysis

Representative samples of the amplicons from all three viruses were chosen for sequencing. Amplicons were purified and sequenced by Sanger’s dideoxy chain termination method. After obtaining the sequencing data, nucleotide homology searches were performed using the BLASTN algorithm on the NCBI database. The sequences were aligned using the MUSCLE algorithm (Edgar, 2004). A phylogenetic tree was constructed to visualize the evolutionary relationships between the sequences using the MEGA X software (Kumar et al., 2018). The maximum-likelihood (ML) criterion is used, and the neighbor-joining method is applied to build the tree.

## Results

### Symptomatology and disease index

**FIGURE 1:**
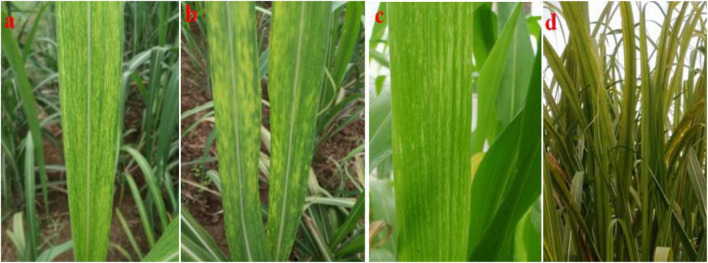
**(a)** Chlorotic areas alternate with green portions on the basal portion of the young leaf due to SCMV infection; **(b)** SCMV-infected leaves show yellow blotches throughout the lamina; **(c)** green inlays alternate with parallel veins due to SCSMV infection; and **(d)** extensive discoloration of the crop canopy due to SCBV infection.

**FIGURE 2:**
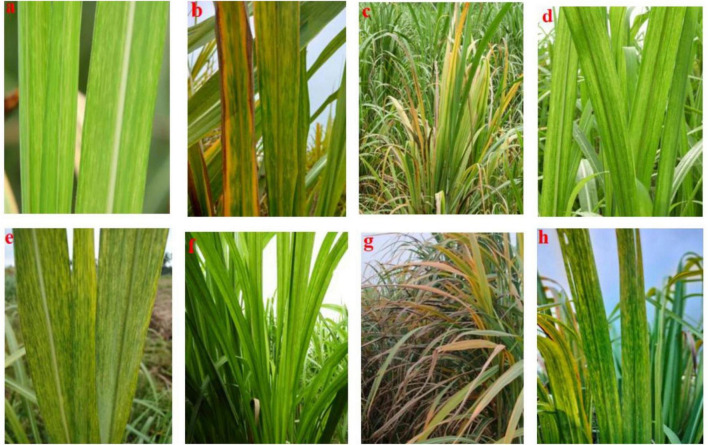
Mosaic symptoms varied from cultivar to cultivar: **(a)** chlorotic areas on pale green lamina (87A 298); **(b)** systemic yellowing and marginal drying of leaf lamina (Co 86032); **(c)** systemic yellowing, drying, and stunting (2009A 107); **(d)** pale yellow-green leaves (Co 997); **(e)** chlorotic streaks expand to large chlorotic patches (2009A 107); **(f)** chlorotic streaks (93A 145); **(g)** systemic yellowing and complete drying (2015A 93); and **(h)** yellow chlorotic areas on green lamina (2018A 166).

**FIGURE 3:**
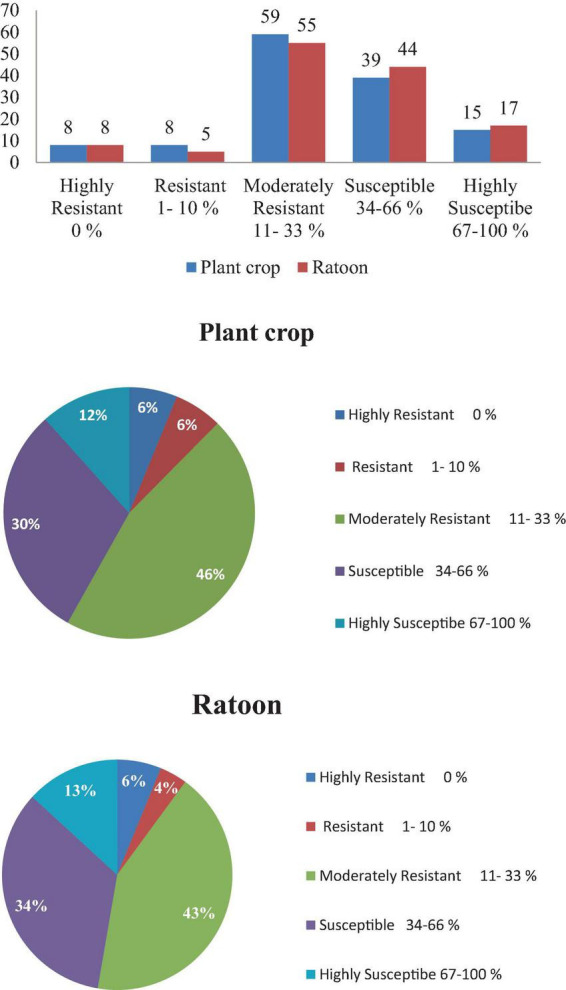
Categorization of sugarcane genotypes into different resistance grades based on field reaction to mosaic under plant and ratoon crop situations.

Screening of 129 elite sugarcane genotypes for broad-based resistance to mosaic was taken up in both plant crop and ratoon under natural field conditions, along with a susceptible check, 2009A 107, at the experimental field of RARS, Anakapalli. Among the tested (129) genotypes, 8 genotypes (2017A 553, 2017A 416, 2017A 517, 2016A 379, Co7717, Co S 767, Baragua, and Co 13034) were scored as highly resistant (grade 1) with null disease index, eight genotypes (2017A 340, 2016A 381, Co 975, Co 1148, Co Se 95422, BO 91, Co J 64, and Co Lk 15206) were scored as resistant (grade 2), 59 genotypes were scored as moderately resistant (grade 3), 39 genotypes were scored as susceptible (grade 4), and 15 genotypes were scored as highly susceptible (grade 5) to mosaic disease in plant crop (Table 2). The susceptible check, 2009A 107, showed 100% incidence. In the ratoon crop, 8 genotypes showed a highly resistant reaction, 5 genotypes showed a resistant reaction, 55 genotypes showed a moderately resistant reaction, 44 genotypes showed a susceptible reaction, and 17 genotypes showed a highly susceptible reaction (Table 2). The 129 genotypes have shown a wide range in the phenotypic expression of mosaic. The tested genotypes exhibited typical mosaic symptoms between 30 and 250 days after planting. Susceptible genotypes produced variable patterns of mosaic symptoms (Figure 1). The initial symptoms of the disease were characterized by the appearance of chlorotic points with linear distribution in the central portion of the leaf, most commonly at the base, which evolved into typical symptoms such as small chlorotic areas interspersed by green bands throughout the length of the leaf. In the case of highly resistant genotypes, there were no symptoms observed as they were lush green throughout the two cropping seasons. In the case of resistant to moderately resistant genotypes, a mild mosaic of chlorotic patterns to severe mosaic symptoms was observed, whereas in the case of highly susceptible genotypes, systemic yellowing, marginal drying of leaves, and severe stunting were observed. In the case of leaf flecks, under field conditions, the affected genotypes exhibited pronounced flecks, chlorotic stripes, mottling, and stunted growth. The disease started as intense white, isolated flecks on the lamina, followed by the spreading of flecks to the top two to three leaves. Later, flecks became severe, with yellowish discoloration on the lamina followed by complete reddening and early drying of the leaves. In all of the genotypes tested, SCSMV was found to be more prevalent than SCMV and SCBV. The tested genotypes exhibited mosaic symptoms, with disease index ranging from 5.50 to 100 and 6.75 to 100% during the 2021–2022 and 2022–2023 crop growing seasons, respectively. Categorization of sugarcane genotypes into different resistance grades based on field reaction to mosaic under plant and ratoon crop situations (Figures 2, 3). Reddening of mature old leaves is the most commonly observed symptom in the genotype 2009A 107. Mild to intense flecks on old leaves were observed in the cultivar 87A 298. Most of the genotypes, 2003V 46, Co 86032, 81V 48, and 2015A 311, exhibited mild to severe white flecks as an initial symptom in the terminal portion of the leaf; later, these resulted in complete mottling of the leaves.

**TABLE 2:** Identification of sugarcane genotypes for their broad-based resistance against mosaic and leaf fleck diseases during 2021–2022 (plant crop) and 2022–2023 (ratoon crop) seasons.

S. no.	Accession no.	Plant crop						Ratoon						
		Days to symptom initiation	DI (%)	DR	RT-PCR/PCR			Days to symptom initiation	DI (%)	% increase over plant crop	DR	RT-PCR/PCR		
					SCMV	SCSMV	SCBV					SCMV	SCSMV	SCBV
1	2017A 553	–	0.00	HR	−	−	−	–	0.00	0	HR	−	−	−
2	2017A 36	50–55	22.06	MR	−	+	−	45–50	26.47	16.67	MR	−	+	−
3	2017A 65	50–55	18.03	MR	+	−	−	45–50	19.67	8.33	MR	+	−	−
4	2017A 103	50–55	30.00	MR	−	+	−	50–55	34.00	6.25	S	−	+	−
5	2017A 191	85–90	12.28	MR	−	+	+	75–80	19.30	36.36	MR	−	+	+
6	2017A 196	70–80	26.92	MR	+	+	−	70–80	33.69	17.65	S	+	+	−
7	2017A 205	85–90	34.50	S	−	−	+	70–75	38.18	14.29	S	−	−	+
8	2017A 253	45–50	19.05	MR	−	+	−	45–50	19.05	0.00	MR	−	+	−
9	2017A 268	45–50	17.65	MR	+	+	−	45–50	23.53	25.00	MR	+	+	−
10	2017A 340	–	0.00	R	−	+	−	–	0.00	5.75	R	−	+	−
11	2017A 405	45–50	49.02	S	+	+	+	40–45	50.98	3.85	S	+	+	+
12	2017A 408	45–50	51.06	S	+	−	−	40–45	53.19	4.00	S	+	−	−
13	2017A 416	–	0.00	HR	−	−	−	–	0.00	0.00	HR	−	−	−
14	2017A 457	45–50	53.19	S	+	−	−	45–50	55.32	3.85	S	+	−	−
15	2017A 497	35–40	66.67	S	−	+	+	35–40	68.89	3.23	HS	−	+	+
16	2017A 68	55–60	23.08	MR	+	−	+	55–60	25.00	7.69	MR	+	−	+
17	2017A 73	75–80	20.83	MR	−	+	−	75–80	31.25	33.33	MR	−	+	−
18	2017A 94	75–80	14.81	MR	−	−	+	75–80	16.67	11.11	MR	−	−	+
19	2017A 187	115–120	25.58	MR	−	−	+	100–105	27.91	8.33	MR	−	−	+
20	2017A 236	175–180	25.00	MR	−	−	+	95–100	28.85	13.33	MR	−	−	+
21	2017A 269	85–90	19.15	MR	+	−	−	80–85	25.53	25.00	MR	+	−	−
22	2017A 313	85–90	19.15	MR	+	−	−	80–85	27.66	30.77	MR	+	−	−
23	2017A 351	85–90	15.79	MR	−	+	−	85–90	21.05	25.00	MR	−	+	−
24	2017A 429	55–60	15.56	MR	+	−	−	45–50	31.11	50.00	MR	+	−	−
25	2017A 517	–	0.00	HR	−	−	−	–	0.00	0.00	HR	−	−	−
26	2018A 6	35–40	20.45	HR	−	+	−	35–40	20.45	0.00	MR	−	+	−
27	2018A 8	35–40	29.17	MR	−	−	+	35–40	31.25	6.67	MR	−	−	+
28	2018A 13	45–50	30.00	MR	+	−	−	45–50	35.00	14.29	S	+	−	−
29	2018A 30	45–50	27.27	MR	−	+	−	45–50	59.09	53.85	S	−	+	−
30	2018A 31	85–90	18.60	MR	−	−	+	80–85	23.26	20.00	MR	−	−	+
31	2018A 33	45–50	11.11	MR	−	+	−	45–50	20.00	44.44	MR	−	+	−
32	2018A 55	55–60	16.28	MR	+	−	−	45–50	20.93	22.22	MR	+	−	−
33	2018A 212	115–120	17.54	MR	+	+	−	100–105	22.81	23.08	MR	+	+	−
34	2018A 107	85–90	39.53	S	−	+	−	40–45	44.19	10.53	S	−	+	−
35	2018A 122	45–50	33.33	S	−	−	+	40–45	46.67	28.57	S	−	−	+
36	2018A 130	45–50	33.33	S	−	−	+	40–45	40.00	16.67	S	−	−	+
37	2018A 133	45–50	37.50	S	+	−	−	40–45	56.25	33.33	S	+	−	−
38	2018A 134	45–50	28.57	MR	−	−	+	40–45	37.14	23.08	S	−	−	+
39	2018A 135	55–60	19.61	MR	+	−	+	45–50	27.45	28.57	MR	+	−	+
40	2018A 137	90–95	26.79	MR	−	+	−	85–90	28.57	6.25	MR	−	+	−
41	2018A 141	55–60	28.00	MR	+	−	−	45–50	32.00	12.50	MR	+	−	−
42	2018A 152	175–180	24.39	MR	+	−	−	145–150	31.71	23.08	MR	+	−	−
43	2018A 144	175–180	18.60	MR	−	−	+	145–150	30.23	38.46	MR	−	−	+
44	2018A 157	85–90	20.00	MR	−	+	−	75–80	30.00	33.33	MR	−	+	−
45	2018A 161	115–120	15.38	MR	−	+	−	95–100	17.31	11.11	MR	−	+	−
46	2018A 166	115–120	27.27	MR	+	−	−	115–120	31.82	14.29	MR	+	−	−
47	2018A 171	85–90	19.61	MR	−	+	−	85–90	29.41	33.33	MR	−	+	−
48	2018A 174	55–60	34.65	S	−	−	+	45–50	36.73	11.11	S	−	−	+
49	2018A 175	55–60	18.87	MR	+	−	−	55–60	26.42	28.57	MR	+	−	−
50	2018A 185	85–90	16.92	MR	−	+	−	80–85	23.08	26.67	MR	−	+	−
51	2018A 188	85–90	12.24	MR	−	−	+	75–80	28.57	57.14	MR	−	−	+
52	2018A 190	60–65	25.00	MR	−	+	−	55–60	29.55	15.38	MR	−	+	−
53	2018A 195	55–60	35.56	S	−	−	+	55–60	37.78	5.88	S	−	−	+
54	2018A 196	45–50	36.96	S	+	+	−	45–50	39.13	5.56	S	+	+	−
55	2018A 202	45–50	35.00	S	+	+	−	40–45	45.00	22.22	S	+	+	−
56	2018A 203	45–50	34.61	S	−	+	−	40–45	39.13	16.67	S	−	+	−
57	2016A 379	–	0.00	HR	−	−	−	–	0.00	0.00	HR	−	−	−
58	2016A 381	–	0.00	R	+	−	−	–	0.00	5.50	R	+	−	−
59	2016A 385	175–180	25.00	MR	+	−	−	165–170	27.08	7.69	MR	+	−	−
60	2016A 395	115–120	17.78	MR	−	+	−	115–120	22.22	20.00	MR	−	+	−
61	2016A 642	175–180	17.07	MR	−	−	+	145–150	19.51	12.50	MR	−	−	+
62	2016A 674	220–225	15.91	MR	−	−	+	175–180	20.45	22.22	MR	−	−	+
63	2016A 680	175–180	19.51	MR	−	−	+	155–160	24.39	20.00	MR	−	−	+
64	2016A 685	115–120	37.04	S	−	+	−	85–90	44.44	16.67	S	−	+	−
65	2016A 719	115–120	41.67	S	+	−	−	85–90	44.44	6.25	S	+	−	−
66	2016A 759	115–120	40.54	S	+	−	−	95–100	48.65	16.67	S	+	−	−
67	2016A 59	85–90	36.36	S	+	−	−	85–90	36.36	0.00	S	+	−	−
68	2016A 254	175–180	17.78	MR	−	+	−	155–160	22.22	20.00	MR	−	+	−
69	2016A 255	85–90	35.56	S	−	+	−	75–80	40.00	11.11	S	−	+	−
70	2016A 484	235–240	46.15	S	−	+	−	215–220	51.28	10.00	S	−	+	−
71	2016A 503	175–180	35.71	S	−	−	+	155–160	40.48	11.76	S	−	−	+
72	2016A 575	115–120	29.79	MR	−	+	−	95–100	31.91	6.67	MR	−	+	−
73	2016A 580	145–150	21.28	MR	−	+	+	135–140	25.53	16.67	MR	−	+	+
74	2016A 592	85–90	22.64	MR	+	−	−	80–85	28.30	20.00	MR	+	−	−
75	2016A 600	175–180	14.00	MR	+	−	−	155–160	22.00	36.36	MR	+	−	−
76	2016A 664	245–250	21.43	MR	−	+	+	215–220	21.43	0.00	MR	−	+	+
77	2016A 672	120–130	42.86	S	+	−	−	85–90	45.71	6.25	S	+	−	−
78	2015A 301	175–180	36.73	S	−	+	−	145–150	40.82	10.00	S	−	+	−
79	2015A 308	175–180	15.38	MR	−	+	−	155–160	41.03	62.50	S	−	+	−
80	2015A 309	175–180	16.00	MR	−	−	+	155–160	20.00	20.00	MR	−	−	+
81	2015A 311	115–120	18.60	MR	−	+	−	105–110	20.93	11.11	MR	−	+	−
82	2015A 333	75–80	34.61	S	+	+	+	70–75	34.69	11.76	S	+	+	+
83	81A 99	95–100	25.00	MR	+	−	+	85–90	30.00	16.67	MR	+	−	+
84	93A 145	25–30	90.91	HS	+	+	+	22–25	95.45	4.76	HS	+	+	+
85	97A 85	25–30	73.68	HS	−	−	+	23–25	76.32	3.45	HS	−	−	+
86	2000A 56	35–40	43.24	S	+	−	−	30–35	51.35	15.79	S	+	−	−
87	2001A 63	45–50	43.59	S	+	−	−	40–45	48.72	10.53	S	+	−	−
88	2003V 46	145–150	40.54	S	−	−	+	135–140	43.24	6.25	S	−	−	+
89	2003A 255	85–90	93.33	HS	+	+	+	80–85	93.33	0.00	HS	+	+	+
90	2004A 55	85–90	65.63	HS	+	−	+	80–85	68.75	4.55	HS	+	−	+
91	Co 7219	55–60	70.37	HS	+	−	+	50–55	74.07	5.00	HS	+	−	+
92	2006A 64	55–60	68.97	HS	−	−	+	50–55	72.41	4.76	HS	−	−	+
93	2006A 102	55–60	48.78	S	−	+	−	55–60	51.22	4.76	S	−	+	−
94	2006A 223	75–80	50.00	S	+	+	−	70–75	55.56	10.00	S	+	+	−
95	2010A 229	55–60	36.00	S	−	+	+	55–60	56.00	42.86	S	−	+	+
96	CoA 7602	55–60	36.77	S	+	−	+	45–50	38.46	20.00	S	+	−	+
97	Co 86032	115–120	42.86	S	−	+	−	110–115	45.71	6.25	S	−	+	−
98	98A 163	75–80	45.45	S	+	−	−	70–75	48.48	6.25	S	+	−	−
99	2000A 225	85–90	37.50	S	+	−	−	70–75	46.88	20.00	S	+	−	−
100	Co 997	35–40	89.74	HS	+	+	−	30–35	92.31	2.78	HS	+	+	−
101	CoC 671	45–50	95.12	HS	+	+	−	40–45	97.56	2.50	HS	+	+	−
102	Co 419	45–50	37.50	S	+	−	−	45–50	40.00	6.25	S	+	−	−
103	Co 975	115–120	8.51	R	−	+	−	115–120	10.64	20.00	MR	−	+	−
104	Co 1148	175–180	6.25	R	−	+	−	175–180	8.33	25.00	R	−	+	−
105	BO 91	–	5.50	R	−	+	−	–	6.75	18.51	R	−	+	−
106	Co J 64	–	8.50	R	+	−	−	–	9.50	10.52	R	+	−	−
107	Co7717	–	0.00	HR	−	−	−	–	0.00	0.00	HR	−	−	−
108	Co S 767	–	0.00	HR	−	−	−	–	0.00	0.00	HR	−	−	−
109	Baragua	–	0.00	HR	−	−	−	–	0.00	0.00	HR	−	−	−
110	Co 62399	85–90	52.08	S	−	+	−	80–85	60.42	13.79	S	−	+	−
111	Co 86002	175–180	36.59	S	+	−	−	155–160	39.02	6.25	S	+	−	−
112	CoS 8436	145–150	39.13	S	−	+	+	135–140	43.48	10.00	S	−	+	+
113	Co Se 95422	–	10.00	R	+	−	−	–	11.50	0.00	R	+	−	−
114	Co 09022	115–120	13.95	MR	−	−	+	115–120	16.28	14.29	MR	−	−	+
115	83V 15	75–80	81.63	HS	−	+	+	65–70	83.67	2.44	HS	−	+	+
116	Co 15024	85–90	12.50	MR	−	+	−	65–70	22.92	45.45	MR	−	+	−
117	Co 15026	85–90	12.24	MR	+	−	−	65–70	30.61	60.00	MR	+	−	−
118	Co 15027	85–90	11.63	MR	−	+	−	65–70	18.60	37.50	MR	−	+	-
119	Co 12029	55–60	37.50	S	+	+	−	50–55	41.67	10.00	S	+	+	−
120	Co 13034	–	0.00	HR	−	−	−	–	0.00	0.00	HR	−	−	−
121	Co 14034	115–120	87.88	HS	+	+	−	105–110	90.91	3.33	HS	+	+	−
122	CoS 08279	115–120	12.20	MR	+	−	−	105–110	19.51	37.50	MR	+	−	−
123	87A 298	55–60	88.89	HS	+	+	+	55–60	91.11	2.44	HS	+	+	+
124	Co Lk 14203	85–90	64.10	HS	−	+	+	80–85	71.79	10.71	HS	−	+	+
125	Co 86002	175–180	17.78	MR	+	−	−	155–160	31.11	42.86	MR	+	−	−
126	Co Lk 15206	175–180	8.70	R	−	+	−	160–165	13.04	33.33	MR	−	+	−
127	Khakai	175–180	65.22	HS	+	+	−	145–150	69.57	6.25	HS	+	+	−
128	CoC 19337	95–100	83.33	HS	−	+	+	85–90	85.71	2.78	HS	−	+	+
129	2009 A 107*	25–30	100.00	HS	+	+	+	15–18	100.00	0	HS	+	+	+

*Susceptible check. DI, Disease incidence; DR, Disease reaction.

### RT-PCR and PCR assays

**FIGURE 4:**
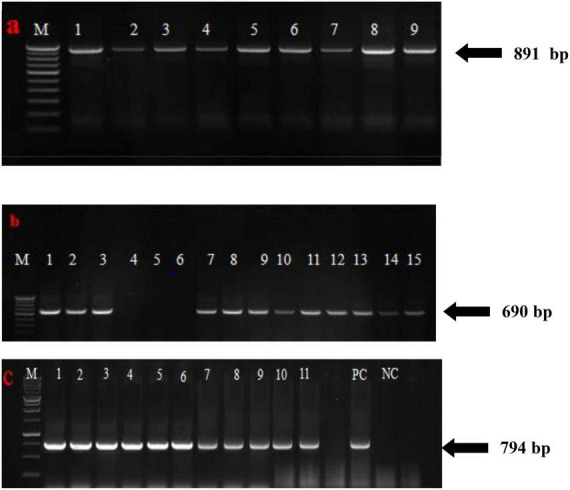
RT-PCR and PCR amplification of the coat protein gene and RT-RNase H region of **(a)** SCMV, **(b)** SCSMV, and **(c)** SCBV from mosaic and leaf fleck affected sugarcane genotypes, respectively (numbers 1, 2, 3,… indicate positive amplification from mosaic and leaf fleck susceptible genotypes).

Field resistance of sugarcane genotypes based on visual grading was further confirmed by RT-PCR/PCR detection of SCMV, SCSMV, and SCBV in symptomatic and asymptomatic leaf samples from 129 sugarcane genotypes. The 891, 690, and 794 bp expected fragments of the SCMV and SCSMV coat protein genes and the RT-RNase H region gene were not amplified in the eight samples obtained from sugarcane genotypes, which were graded as highly resistant (grade 1) to all three viruses in the plant crop. These expected amplicons were present in all resistant to highly susceptible tested genotypes rated as grades 2–5 (Figure 4). Genotypes that were scored as highly resistant under field conditions in the plant crop were further tested under greenhouse conditions in the next season by artificial inoculation 45 days after planting. The results of the study clearly showed that all the genotypes rated as grade 1 under field conditions remained highly resistant under greenhouse evaluation except 2017A 340 and 2016A 381.

The genotypes 2009A 107, 93A 145, 87A 298, 206A 223, 2018 202, 2018A 107, 2017A 497, 2018A 135, Co 997, Khakai, CoC 671, Co 62399, and Co 86032 displayed severe mosaic symptoms and could be quickly identified as diseased even from a few yards away. Younger leaves, often the center leaf, in particular, displayed severe mosaic symptoms such as green streaks and mosaic symptoms throughout the leaf lamina in all tested genotypes. The typical mosaic green inlays/streaks were found to be confined to the proximal part of the leaf lamina in various genotypes, including 2000A 225, 98A 163, 2010A 229, and CoLk 14203, whereas the distal end was devoid of the mosaic symptoms.

At 2–3 months of age, the sugarcane genotypes 2016A 254, 2015A 309, 81A 99, 2006A 223, Co 86032, Co 15024, Co 740, and CoC 671 were reported to exhibit mosaic symptoms; however, when the crop was checked again at sixth and seventh months, there were no such symptoms. The symptoms progressively subsided as the leaves grew older. Similar kinds of gradual disappearing symptoms with the aging of the leaves were observed by Balamuralikrishnan et al. (2003). Therefore, it has been determined that as the crop ages, the mosaic virus titer drops. However, it was found that the majority of the genotypes under study had symptoms throughout their growth phase and did not fully recover from mosaic until the end of the cropping seasons. From the second to the ninth month of crop age, Co se 95422, BO 91, and CoJ 64 were all lush green and free of any evidence of mosaic, but RT-PCR assays showed the presence of virus infection. RT-PCR assays for the genotypes 2017A 340 and 2016A 381 showed the absence of virus infection in plant crops. The same genotypes showed the presence of SCMV and SCSMV infection in RT-PCR assays in ratoon even though they are asymptomatic and may act as carriers of viruses (Figure 4).

### Phylogenetic analysis

**FIGURE 5:**
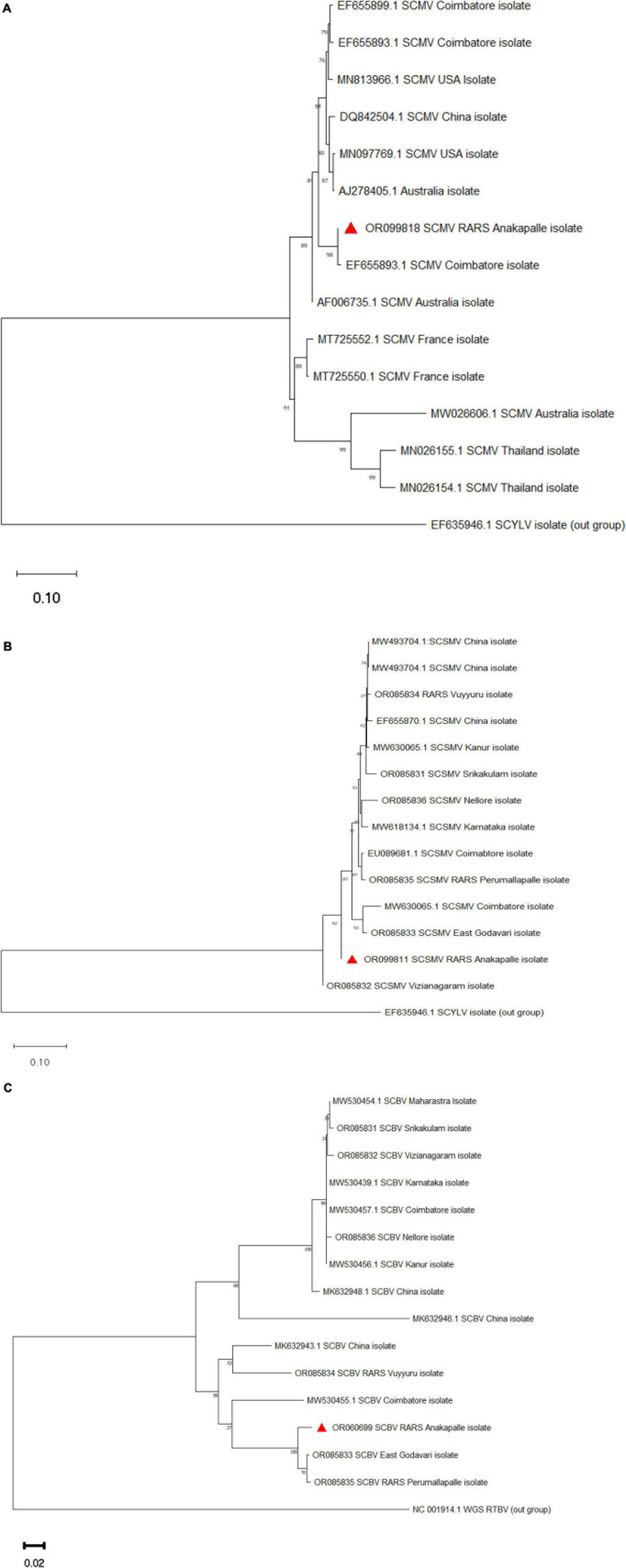
Phylogenetic analysis of nucleotide sequences of **(A)** SCMV, **(B)** SCSMV, and **(C)** SCBV along with retrieved sequences from NCBI. The scale bar represents 0.10 and 0.02 substitutions per nucleotide position for SCMV, SCSMV, and SCBV sequences, respectively (the red triangle indicates the current study virus isolate).

The representative nucleotide sequences of SCMV, SCSMV, and SCBV were subjected to BLAST analysis and shared maximum nucleotide homology with most of the Indian isolates. Phylogenetic analysis was performed with the nucleotide sequences of SCMV, SCSMV, and SCBV, along with corresponding sequences available in NCBI. The isolates of SCMV, SCSMV, and SCBV clustered with most of the other Indian and foreign isolates (Figure 5).

## Discussion

Mosaic and leaf fleck diseases of sugarcane are systemic diseases most commonly transmitted through infected seed cane. Bagyalakshmi et al. (2019) reported that mixed infections of SCMV and SCSMV lead to severe degeneration of cv. CoJ 64. Drastic reductions in cane growth and yield due to the mixed infection of mosaic viruses and their association with SCBV in causing varietal degeneration of popular cultivated varieties were reported in India (Singh et al., 2003; Viswanathan and Balamuralikrishnan, 2005; Viswanathan, 2016). Despite the serious prevalence of mosaic and leaf fleck diseases of sugarcane in India, there have not been significant efforts to identify the resistance sources against these diseases. Identifying and developing resistant cultivars is crucial for managing and minimizing the impact of diseases on sugarcane crops.

The most economical, practical, and effective strategy for controlling mosaic and leaf fleck diseases is to identify, develop, and cultivate sugarcane varieties that are resistant to the diseases. Resistance to multiple viruses is crucial because it can provide broader protection against different strains of the disease and reduce the chances of new infections or virus evolution. Breeding disease-resistant cultivars begins with the exploration and use of a genetic resource. Furthermore, systematic studies are required to understand the single or compound infection of viruses to address the resistance to mosaic and leaf fleck in India. In the current study, resistance to SCMV and SCSMV, the two major causes of mosaic disease, and SCBV, the cause of leaf fleck disease, was identified in 129 new elite sugarcane varieties/clones in both plant and ratoon crops. The tested genotypes exhibited typical mosaic symptoms varying with disease index ranging from 5.50 to 100 and 6.75 to 100% in plant and ratoon crops, respectively. Similar results were reported by Bagyalakshmi and Viswanathan (2020), who reported that seven genotypes were found to be free of mosaic viruses out of 210 genotypes tested. Similarly, Li et al. (2018) and Li et al. (2019) reported that 23 genotypes were found to be resistant to or moderately resistant to mosaic among the 71 genotypes tested. Silva et al. (2015) identified that 22 genotypes were found to be resistant to SCMV among 79 sugarcane genotypes, and resistance was confirmed with plate-trapped antibody ELISA. Disease index, as well as symptom expression, was higher in the case of the ratoon crop. Irrespective of the genotype, disease severity increased in ratoon crops. The percentage of mosaic incidence was high in the ratoon crop when compared to the plant crop. The percentage of disease increased in the ratoon crop compared to the plant crop and varied among the genotypes from 0 to 60%. Similar results were reported by Guadie et al. (2019), who stated that the percentage of mosaic disease increase in the ratoon crop ranged from 18 to 83%. Moreover, symptom expression was earlier in sugarcane genotypes in the ratoon crop than in the plant crop. Complete yellowing of leaves and stunting at an early stage were observed in severely infected genotypes in the ratoon crop.

To confirm the presence or absence of the virus, RT-PCR/PCR detection was utilized. Eight genotypes showed tolerance to all three viruses. These findings offer a superior resistance source for the efficient prevention and management of mosaic and leaf fleck diseases, and they could be used as a guide for commercial cultivars. Two viruses, SCMV and SCSMV, are the two major viruses that can cause mosaic disease, and SCSMV has become a major pathogen responsible for mosaic disease in the sugarcane-growing regions of India (Viswanathan and Rao, 2011). Furthermore, the study revealed that plants that appear healthy and show no visible symptoms (asymptomatic plants) are not necessarily free from mosaic and leaf fleck viruses. Despite not showing symptoms, these plants may still carry the viruses, which are in a latent stage. However, these plants serve as hidden sources of the virus and can contribute to the rapid spread of the disease under field conditions. Asymptomatic plants, along with potential vectors, can significantly worsen the incidence of vector-borne plant diseases. The presence of asymptomatic plants and vectors in the same agricultural environment can lead to increased disease transmission. Within just two growing seasons, new clones become infected with the viruses and start showing symptoms of mosaic and leaf fleck diseases with varying degrees of severity.

The resistance mechanism that plants have against viruses can be compromised by several factors. These include the presence of new virus strains, favorable environmental conditions that aid in virus multiplication and infection, the presence of virus-carrying vectors nearby, and more. Geijskes et al. (2004), Parameswari et al. (2013), and Bagyalakshmi et al. (2019) conducted detailed studies on mosaic and leaf fleck viruses, specifically on the whole genome of viruses SCMV, SCSMV, and SCBV, respectively. They found that negative selection pressure and recombination hotspots throughout the genome of both viruses may contribute to the viruses’ ability to successfully infect plants, develop diseases, and adapt to different environmental conditions. The negative selection pressure could help maintain genetic stability in the viral genome. The Hc-Pro (helper component proteinase) gene is known to govern RNA silencing suppressor (RSS) activity in members of the Potyviridae family, which includes mosaic viruses. However, recent research (Bagyalakshmi and Viswanathan, 2020) demonstrated that the P1 gene in SCSMV has RSS activity. This suggests that the P1 gene might play a significant role in suppressing the host’s resistance mechanism against the virus. In certain instances, SCSMV appears to dominate over SCMV and SCBV in sugarcane plants across different states in India. qRT-PCR assays show that SCSMV has a much higher virus titer than SCMV in infected plants (Viswanathan and Karuppaiah, 2010; Bagyalakshmi et al., 2019).

Among the three viruses, SCSMV was found to be the most dominant, with the distribution frequency being as follows: SCSMV > SCBV > SCMV. Infection from a combination of viruses has the potential to be more virulent and harmful than infection from a single virus. According to reports, a crop suffers more damage from a combined SCMV and SrMV infection than from a solo infection with one of these viruses (Xu et al., 2008). The combined infection may also function synergistically to speed up the progression of the disease in elite cultivars and cause sugarcane degeneration (Viswanathan, 2016).

## Conclusion

The most practical and economical method for managing any viral disease in a crop that is propagated vegetatively, such as sugarcane, is host-plant resistance. Despite the fact that very few researchers have found the origins of mosaic and leaf fleck resistance to date, the majority of their investigations have only evaluated a small number of genotypes. Contrarily, *via* our efforts, we were able to identify eight tolerant genotypes with widespread resistance to mosaic and leaf fleck, including 2017A 553, 2017A 416, 2017A 517, 2016A 379, Co7717, Co S 767, Baragua, and Co 13034, which may be used as donors for breeding new varieties that are resistant to the mosaic and leaf fleck viruses. In the current investigation, we also found significant disease susceptibility in sugarcane genotypes and widespread disease occurrence in the majority of genotypes. The prevalence of SCSMV was found to be greater than that of SCMV and SCBV. All three viruses can co-infect, causing severe symptom manifestation and degeneration. The screened germplasms may offer prospective sources of resistance for the development of resistant cultivars to the sugarcane mosaic and leaf fleck diseases.

## Data availability statement

The datasets presented in this study can be found in online repositories. The names of the repository/repositories and accession number(s) can be found below: https://www.ncbi.nlm.nih.gov/genbank/, OR099818, OR099811, and OR060699.

## Author contributions

GVa: Investigation, Writing - original draft. VMK: Supervision. PKV: Conceptualization, Methodology, Resources. BB: Visualization. GVi: Visualization.
